# The role of cervical mediastinoscopy in Nigerian thoracic surgical practice

**DOI:** 10.11604/pamj.2016.24.135.7668

**Published:** 2016-06-10

**Authors:** Bode Falase, Mgbajah Ogadinma, Adetinuwe Majekodunmi, Barakat Nimasahun, Olufunke Adeyeye

**Affiliations:** 1 Cardiothoracic Division, Department of Surgery, Lagos State University College of Medicine, Lagos State University Teaching Hospital, Lagos, Nigeria; 2 Department of Anaesthesia, Lagos State University College of Medicine, Lagos State University Teaching Hospital, Lagos, Nigeria; 3 Department of Paediatrics, Lagos State University College of Medicine, University Teaching Hospital, Lagos, Nigeria; 4 Respiratory Unit, Department of Medicine, Lagos State University College of Medicine, Lagos University Teaching Hospital, Lagos, Nigeria

**Keywords:** Mediastinoscopy, Isolated Mediastinal Lymphadenopathy, Lung Cancer, Nigeria

## Abstract

**Introduction:**

Cervical mediastinoscopy is the gold standard for obtaining histological diagnosis of mediastinal pathology. It has been used for the staging of lung cancer as well as to determine the cause of Isolated Mediastinal Lymphadenopathy. There is very limited evidence in the literature of its use in Nigeria to assess mediastinal pathology. The aim of this study was to describe our institutional experience with cervical mediastinoscopy.

**Methods:**

This study was a retrospective analysis of 40 patients that underwent cervical mediastinoscopy in our institution between March 2007 and February 2013.

**Results:**

The indication for Cervical Mediastinoscopy was Isolated Mediastinal Lymphadenopathy in 24 patients (60%) and lung cancer staging in 16 patient (40%). The mean age of the patients was 52.7 + 15.1 years. There were 21 females (52.5%) and 19 males (47.5%). The most commonly biopsied lymph nodes were level 4 in 35 patients (87.5%) and level 7 in 21 patients (52.5%). Malignant diagnosis was made in 16 (66.7%) patients with Isolated Mediastinal Lymphadenopathy and in 13 (81.3%) patients staged for lung cancer. Hospital stay was less than 24 hours in all patients and there were no complications.

**Conclusion:**

Cervical Mediastinoscopy is available in Nigeria and has been performed in our institution with high diagnostic yield and no complications. Its increased use, along with the development of other mediastinal biopsy techniques is advocated to increase tissue biopsy of mediastinal pathology, especially for lung cancer and isolated mediastinal lymphadenopathy.

## Introduction

Mediastinal lymphadenopathy may occur either as suspected mediastinal extension of lung cancer or isolated mediastinal lymphadenopathy (IML). Histological diagnosis is to establish whether nodal disease exists or the type of mediastinal pathology present in IML as this determines the prognosis and selection of appropriate therapy [1–5]. Localization of mediastinal lymphadenopathy is usually by Computerized Tomography (CT) or Positron Emission Tomography (PET) scan [1] and a variety of methods are available to obtain specimen from involved lymph nodes. The traditional means of mediastinal biopsy has been by cervical mediastinoscopy, the gold standard since its introduction in 1959 [1, 6, 7]. Recently, less invasive methods of mediastinal biopsy like Endobronchial ultrasound guided transbronchial node aspiration (EBUS-TBNA) and Endoscopic esophageal ultrasound guided fine needle aspiration (EUS-FNA) have been developed as viable alternatives [1, 8, 9]. The incidence of lung cancer in Nigeria is as high as 7.9/100,000 and the presentation is often late with mediastinal involvement which precludes surgical resection [10]. IML also occurs, secondary to various pathologies including tuberculosis, sarcoidosis and lymphoma [3, 4, 11]. There is however only one report in the English literature of mediastinal biopsy in Nigeria [12] thus suggesting that mediastinal biopsy techniques are underutilized in the assessment of mediastinal pathology. The aim of this study was to describe the experience in our institution of mediastinal biopsy with cervical mediastinoscopy.

## Methods

### Institutional setting

The study was conducted in a tertiary health centre located in an urban city in South-West Nigeria. The tertiary centre is one of two such centres in the city responsible for providing cardiothoracic care for an estimated 16 million people resident in the state. The facility has a Cardiothoracic Division with facilities for mediastinoscopy. Mediastinal lymphadenopathy was diagnosed by contrast CT scan in patients being evaluated for lung cancer or IML. Mediastinal lymphadenopathy was defined as the presence of mediastinal lymph nodes greater than 1cm in the short axis [13]. PET scan is not available in Nigeria so was not used for localization of mediastinal lymphadenopathy. The International Association for the Study of Lung Cancer (IASLC) lymph node station map was used to document involved nodal stations [14]. Patients with mediastinal lymph nodes in stations 2, 4 or 7 which are accessible by conventional cervical mediastinoscopy were scheduled for mediastinoscopy. General Anaesthesia with standard ASA monitoring was instituted with the pulse oximetry probe applied to the right hand for possible compression and obstruction of the innominate artery by the mediastinoscope (Karl Storz, Tuttlingen, Germany). With the patient in the supine position, mediastinoscopy was performed in the standard fashion, via a 4cm suprasternal transverse incision. The skin and platysma were divided by sharp dissection and the strap muscles separated by blunt dissection to access the trachea. The pretracheal fascia was breached and the mediastinal space explored by digital palpation prior to introducing the mediastinoscope. A minimum of three biopsies were taken from each involved lymph node station. If any mediastinal oozing was noted the mediastinum was packed with absorbable haemostatic agents. Following adequate haemostasis and wound closure, patients were observed on the ward overnight and reviewed in clinic with the histology results and appropriate therapy instituted.

### Methods

All patient data was stored on a prospectively maintained Microsoft Access database. Institutional ethics committee approval was obtained for interrogation of the database. The study was a retrospective analysis of data from March 2007 to February 2013. Data was extracted for 40 patients that had undergone cervical mediastinoscopy. Data of patients that had alternate methods of biopsy were excluded. Extracted data included patient demographics, the indication for mediastinoscopy, the nodal stations involved, the histology of biopsied nodes and any complications. Data analysis was done with Microsoft excel 2013. Summary data is presented as mean ± standard deviation, numbers or percentages as appropriate.

## Results

The indication for cervical mediastinoscopy was Isolated Mediastinal Lymphadenopathy (IML) in 24 patients (60%) and staging of lung cancer in 16 patients (40%). The mean age of the patients was 52.7 + 15.1 years. There were 21 females (52.5%) and 19 males (47.5%). The distribution of involved nodal stations was level 4R in 35 patients (87.5%), level 7 in 21 patients (52.5%), level 4L in 17 patients (42.5%), , level 2L in 5 patients (12.5%), level 2R in 5 patients (12.5%) and 26 patients (65%) had two or more involved lymph node stations. Histological diagnosis was malignant in 29 patients (72.5%) and benign in 11 patients (27.5%). The 29 malignant diagnoses were carcinoma in 14 patients (48.3%), adenocarcinoma in 9 patients (31%), lymphoma in 5 patients (17.2%) and thymoma in 1 patient (3.4%). The 11 benign diagnoses were tuberculosis in 4 patients (36.4%), sarcoidosis in 4 patients (36.4%) and anthracosis in 3 patients (27.3%). The distribution of histological diagnosis according to the indication for mediastinoscopy is as shown in Table 1 (staging for lung cancer) and Table 2 (isolated mediastinal lymphadenopathy). When mediastinoscopy was done to stage lung cancer there was malignant pathology in 13 (81.3%) patients and benign pathology in 3 (18.7%) patients. For the patients with IML 16 (66.7%) patients had malignant pathologies and 8 (33.3%) had benign pathologies. All the patients were discharged home within 24 hours of surgery. There were no complications. All patients were seen in clinic where the histology results were reviewed and appropriate therapy instituted.

**Table 1:** Histological diagnosis in patients staged for lung cancer

Histology	Number	Total (%)
		
**Malignant**		**13 (81.3)**
Carcinoma	5	
Carcinoma-bronchoalveolar	2	
Carcinoma-squamous	2	
Adenocarcinoma	2	
Adenocarcinoma-colonic	1	
Lymphoma -Hodgkin's	1	
		
**Benign**		**3 (18.7)**
Tuberculosis	2	
Anthracosis	1	
		**16**

**Table 2:** Histological diagnosis in patients with isolated mediastinal lymphadenopathy

Histology	Number	Total (%)
		
***Malignant***		**16 (66.7)**
Adenocarcinoma	4	
Adenocarcinoma-breast	1	
Adenocarcinoma-prostate	1	
Carcinoma	4	
Carcinoma-squamous	1	
Lymphoma-Non-Hodgkin's	4	
Thymoma	1	
		
**Benign**		**8 (33.3)**
Sarcoidosis	4	
Tuberculosis	2	
Anthracosis	2	
		**24**

## Discussion

Mediastinal lymphadenopathy can either be due to malignant or benign disease. The most common reason for malignant disease, as in our series, is lung cancer. Following localization of disease with CT-PET scan, histological diagnosis is mandatory to direct appropriate therapy [15]. Guidelines have been established which advice on the need to establish histological diagnosis of suspected mediastinal extension of lung cancer to direct therapy [16]. It is not clear how closely these guidelines are adhered to in Nigeria and West Africa. The basic tools to establish histological diagnosis are often lacking. CT is not yet widely available and there is no PET scanner in Nigeria. Even where CT is available, physicians are often not able to access the mediastinum to obtain tissue for histological diagnosis [17, 18]. Modalities like Mediastinoscopy, Video Assisted Thoracic Surgery, TBNA, EBUS-TBNA and EUS-FNA which are commonly available in industrialized nations need to be used in the developing world to increase the diagnostic yield of biopsy for mediastinal pathology.

Mediastinoscopy is now being practiced less in industrialized nations as TBNA, EBUS-TBNA and EUS-FNA have gained in popularity. These alternate techniques of mediastinal biopsy avoid the need for general anaesthesia and are less invasive than mediastinoscopy. Though the sensitivity of these techniques is high, especially with the introduction of ultrasound image guidance, the Achilles heel of these techniques is their low negative predictive value. A negative test therefore does not exclude the presence of a pathological lymph node sois usually still followed by mediastinoscopy [9]. Certainly where all these techniques are available, it reduces the need for mediastinoscopy if the tissue diagnosis can be obtained by less invasive means [8]. In the series by Navani et al the cost of performing EBUS-TBNA was $2,190 and mediastinoscopy was $5,115 [8]. This contrasts sharply with the cost in our institution which is $550 for mediastinoscopy. Though the cost of EBUS-TBNA is cheaper than mediastinoscopy in reports, the startup costs can be prohibitive so the use of mediastinoscopy in suitable patients still remains the best available and relatively affordable option presently in our environment.

In our study, we have shown that mediastinoscopy can be performed safely with minimal complications and a high diagnostic yield but care must however be taken to perform it properly, sampling all accessible lymph node stations. It has been shown, especially for lung cancer, that the more thorough the search for pathological mediastinal lymph nodes, the more accurate is the staging of lung cancer and survival following treatment is better largely because appropriate therapy is being given for the correct stage of disease [19]. In the USA, it has been shown in a large series of 11,668 patients with Non-small cell lung cancer who proceeded to lung resection that only 27% had mediastinoscopy. Of those that had mediastinoscopy, lymph nodes were biopsied in only 46.6% of the patients [20]. Even at the time of lung resection there have been reports that sampling of mediastinal lymph nodes has been inaccurate, leading to wrong staging of disease [21]. This has led to guidelines which have defined the thoroughness of mediastinal lymph node examination, ranging from complete mediastinal lymphadenectomy (the gold standard) to systematic sampling (less accurate) and selective sampling of lymph nodes which is discouraged due to low diagnostic yield [22]. In our series though we opted for selective sampling, mainly to reduce operative time, we took at least three biopsies from selected lymph nodes to increase the diagnostic yield. This appears to have been successful as we obtained tissue for histological diagnosis in all the patients suspected to have lung cancer, confirming N2 disease in 81.3% of the patients thereby sparing them an unnecessary thoracotomy. This is in contrast to other West African series where bronchopulmonary disease has been evaluated without mediastinoscopy. In a recent series from Benin bronchoscopy was used to evaluate patients suspected to have lung cancer. No tissue was obtained in 37.5% of the patients and only 25% of the patients had lung cancer confirmed from bronchoscopic biopsy and histology. The remaining patients required open lung biopsy to confirm the diagnosis of lung cancer [17]. In a series from Gambia, Bah et al obtained tissue diagnosis in only 13% of lung cancer cases and stressed that a great challenge to cancer care in West Africa is poor documentation and difficulties in obtaining tissue diagnosis [23]. Increased use of mediastinoscopy could increase the diagnosis of malignant pathology in the mediastinum as shown in our series where 72.5% of the histological diagnoses were of malignant pathology. Mediastinoscopy has also been shown to useful in obtaining tissue diagnosis of IML. Similar to our series, diagnosis of tuberculosis [11], sarcoidosis [3] and lymphoma [4, 5] can be obtained by mediastinoscopy. Though these diagnoses can often be made by other less invasive tests it is useful to establish the diagnosis by mediastinal biopsy in unresolved cases of mediastinal masses as unnecessary thoracotomies can be avoided and appropriate therapy be instituted as opposed to blind therapy [4]. The average morbidity and mortality from cervical mediastinoscopy are 2% to 3% and 0.3% to 0.8% respectively [24, 25]. Complications are due to damage mediastinal structures and the most common include pneumothorax, vocal cord dysfunction and haemorrhage which is the most feared complication. Large series have however shown the risk of haemorrhage to be as low as 0.4% [25]. Cervical mediastinoscopy is therefore very safe once the necessary experience has been acquired.

Conventional cervical mediastinoscopy only accesses lymph nodes at levels 2,3,4 and 7 as in this series where the most commonly sampled lymph nodes were at levels 4 and 7. EBUS-TBNA can access levels 1,2,3,4,7,8,10,11 and 12. EUS-FNA can access levels 4, 5, 7, 8 and 9 while VATS can access levels 5 and 6 [14]. These various techniques should therefore be seen as complementary to each other and the specific choice of which to use is driven by the level of the lymph nodes to be sampled [1, 16]. Therefore though the increased use of mediastinoscopy is encouraged, the other mediastinal biopsy techniques also need to be available to complete the armamentarium of tools available for evaluation of mediastinal pathology that presents in Nigeria [18].

## Conclusion

Mediastinoscopy is available in Nigeria and has been performed in our institution with high diagnostic yield and no complications. Its increased use, along with the development of other mediastinal biopsy techniques is advocated to increase tissue biopsy of mediastinal pathology, especially for lung cancer and isolated mediastinal lymphadenopathy.

### What is known about this topic

- Cervical mediastinoscopy is the gold standard for obtaining histological diagnosis of mediastinal pathology;
- It is commonly used for staging of lung cancer and to determine the cause of Isolated Mediastinal Lymphadenopathy;
- There is however limited evidence in the literature of the use of cervical mediastinoscopy in Nigeria.

### What this study adds

- Cervical mediastinoscopy is available in Nigeria;
- It can be performed safely in our environment with high diagnostic yield;
- Increased use of cervical mediastinoscopy is encouraged to improve the staging of lung cancer and improve histological diagnosis of Isolated Mediastinal Lymphadenopathy.
